# Follicular Thyroid Carcinoma in a Developing Country: A 10-Year Retrospective Study

**DOI:** 10.7759/cureus.16594

**Published:** 2021-07-23

**Authors:** Abdul Aziz, Muhammad Qamar Masood, Saadia Sattar, Saira Fatima, Najmul Islam

**Affiliations:** 1 Department of Medicine, Section of Endocrinology, Aga Khan University Hospital, Karachi, PAK; 2 Department of Pathology & Laboratory Medicine, Section of Histopathology, Aga Khan University Hospital, Karachi, PAK

**Keywords:** thyroid carcinoma, follicular thyroid carcinoma, developing country, clinical characteristics, treatment choices

## Abstract

Background

The most common endocrine tumor is thyroid cancer. Follicular thyroid carcinoma (FTC) accounts for 5-10% of all thyroid cancers. Patients with FTC frequently present with more advanced diseases and a higher occurrence of distant metastases because of the propensity of vascular invasion. FTC is mainly treated with surgery while radioactive iodine (RAI) therapy is the main adjuvant therapy according to the American Thyroid Association guidelines.

Methodology

This was a retrospective observational study of FTC patients aged 18 and above conducted at a tertiary care hospital in Karachi from January 01, 2010 to December 31, 2019.

Results

A total of 404 patients with thyroid carcinoma were sorted, of which 40 (10.1%) were FTC cases. Overall, 50% of the patients were in the age group of 41-60 years, and the female-to-male ratio was 1.5:1. The majority of patients (60%) presented with neck swelling, followed by bone and lung metastasis in 20% and compressive symptoms in another 20%. On fine needle aspiration cytology (FNAC), 50% had Bethesda category III-IV nodules while 10% had Bethesda category II. Overall, 50% had a total thyroidectomy while 50% had a lobectomy followed by a completion thyroidectomy. On histopathology, 23 (57.5%) patients had minimally invasive FTC while 17 (42.5%) had widely invasive FTC. A total of 17 (42.5%) patients had received RAI 30-100 mCi while 10 (25%) received more than 100 mCi.

Conclusions

FTC can present with both local or metastatic symptoms. The atypical presentation of metastatic FTC should be considered, diagnosed, and managed early to limit mortality and morbidity. Ultrasound is the best diagnostic investigation of choice followed by FNAC. Surgery is the mainstay of treatment and should be followed by RAI in select cases. Thus, understanding the trend of FTC and proper planning and utilization of the resources will help developing countries in effectively treating the FTC.

## Introduction

The most common endocrine tumor is thyroid cancer, the incidence of which is rising. Thyroid cancer is expected to become the fourth most common cancer in the United States by 2030. The annual incidence rate of thyroid cancer in various parts of the world ranges from 0.5 to 10 cases per 100,000 population [1]. In Pakistan, it accounts for 1.2% of all cancers [2]. Both papillary and follicular carcinoma of the thyroid are collectively referred to as differentiated thyroid cancer [3]. Follicular thyroid carcinoma (FTC) accounts for 5-10% of all thyroid cancers [4,5]. Invasion of the capsule and vessels is the unique feature differentiating follicular carcinomas from follicular adenomas. According to the American Thyroid Association (ATA) guidelines of 2015, FTC is classified into minimally invasive and widely invasive tumors based on the number of vascular and capsular invasions [6]. Patients with FTC frequently present with advanced-stage diseases and a higher incidence of distant metastases because of the propensity of vascular invasion [7,8].

Although there have been numerous studies regarding the diagnosis, treatment, and clinicopathological features of patients with papillary thyroid carcinoma, relatively few address FTC patients across the world. There is no large study exclusively addressing the characteristics of FTC in Pakistan. Hence, the current study aimed to determine the demographic, clinicopathological, and radiological features of FTC. In many developing countries including Pakistan, surgical facilities and radioactive iodine (RAI) therapy are in short supply, therefore understanding the trends of FTC may help developing countries to plan and utilize resources more effectively [9].

## Materials and methods

This was a retrospective observational study from January 01, 2010 to December 31, 2019 conducted in the Department of Medicine at a tertiary care hospital in Karachi. Approval was taken from the Ethical Review Committee (ERC) of our institute (ERC # 2020-4845-10862), and Health Information and Management Services (HIMS) was requested to provide a list of all patients diagnosed with thyroid carcinoma during the study period. Data of all patients aged 18 and above admitted with the diagnosis of thyroid carcinoma were obtained, of which patients who had FTC on histopathology were included in the study. There were a total of 404 patients with thyroid cancers, of which 40 (10.1%) fulfilled our inclusion criteria. All data were obtained from the hospital database originating from computerized medical records and file review. The age, gender, sign and symptoms, and thyroglobulin level at the time of initial presentation were noted and analyzed. The ultrasound features, fine needle aspiration cytology (FNAC) (according to Bethesda classification), type of surgery, and histopathological features were also noted along with the RAI dose (if given). According to the Bethesda classification, FNAC was divided as category I-II for unsatisfactory and benign and category III-IV for atypia of undetermined significance or follicular lesion of undetermined significance and follicular neoplasm or suspicious for a follicular neoplasm. As follicular carcinoma is only definitely diagnosed via histopathology, Bethesda category V and VI were not included in our study. Histopathological features included the type of FTC, size, capsular invasion, lymphovascular invasion, and lymph node involvement. The American Joint Committee on Cancer staging applicable in the cases was recorded. Stata version 14.0 (StataCorp., College Station, TX) was used to analyze the data.

## Results

Table 1 illustrates the general characteristics of the study population. The majority (67.5%) of our patients were above the age of 40, and 50% were in the age group of 41-60 years. The female-to-male ratio was 1.5:1.

**Table 1:** General characteristics of the study population.

Variable	n = 40 (%)
Age (in years)	
18-40	13 (32.5)
41-60	20 (50.0)
61-80	7 (17.5)
Gender	
Male	16 (40.0)
Female	24 (60.0)

Table 2 lists the initial symptoms on presentation. Majority of the patients (60%) presented with neck swelling, followed by compressive symptoms and metastasis. The most common site of metastasis was bone (87.5%) followed by lung (12.5%). The metastasis was more common in males compared to females (31.5% vs. 12.5%).

**Table 2:** Initial presentation and preoperative thyroglobulin.

Symptoms on presentation	n = 40 (%)
Neck swelling	24 (60.0)
Compressive symptoms	8 (20.0)
Metastasis	8 (20.0)
Preoperative thyroglobulin done	6 out of 8 metastatic patients (75)

Table 3 illustrates the FNAC findings. Most patients were in Bethesda category III-IV (50%) while only 10% were in Bethesda Category II.

**Table 3:** Fine needle aspiration cytology.

Fine needle aspiration cytology	n = 40 (%)
Bethesda category II	4 (10.0)
Bethesda category III-IV	20 (50.0)
Not available (initial presentation as metastasis or compressive symptoms)	16 (40.0)

Table 4 lists the type of initial thyroid surgery. Half of the patients had a total thyroidectomy and the remaining had a lobectomy followed by a completion thyroidectomy. The neck lymph node dissection was done in 10 patients, of whom seven had lymph node involvement on histopathology.

**Table 4:** Initial surgery and lymph node dissection.

Type of initial thyroid surgery (thyroidectomy)	n = 40 (%)
Lobectomy followed by completion thyroidectomy	20 (50.0)
Total thyroidectomy	20 (50.0)
Lymph node dissection	
No	30 (75.0)
Yes	10 (25.0)

Table 5 illustrates the histopathological features of FTC. As shown in the table, the predominant type was minimally invasive FTC (57.5%).

**Table 5:** Histopathological features of follicular thyroid carcinoma.

Histopathological features	n = 40 (%)
Type	
Minimally invasive	23 (57.5)
Widely invasive	17 (42.5)
Size	
0-2 cm	4 (10.0)
2.1-4 cm	11 (27.5)
>4 cm	19 (47.5)
Not available	6 (15.0)
Capsular invasion	
No	1 (5)
Yes	39 (95)
Lymphovascular invasion	
No	8 (20.0)
Yes	26 (65.0)
Not available	6 (15.0)
Regional lymph node involvement	
No	3 (7.5)
Yes	7 (17.5)
No lymph node dissection done	30 (75.0)

Table 6 lists the clinical staging and RAI dosage. Overall, 65% of the patients had clinical stage 1 disease and the majority (42.5%) of patients received an RAI dose of 30-100 mCi.

**Table 6:** Clinical staging and radioactive iodine dose in follicular thyroid carcinoma.

	n = 40 (%)
Clinical staging	
Stage 1	26 (65.0)
Stage 2	10 (25.0)
Stage 4	4 (10.0)
Radioactive iodine	
Not given	3 (7.5)
30-100 mCi	17 (42.5)
>100 mCi	10 (25.0)
Not available	10 (25.0)

## Discussion

In our study, 10.1% of the patients had FTC among all the thyroid carcinoma patients similar to the trends reported from the United States, where 9% of thyroid carcinomas were follicular carcinomas between 1973 and 2002 [10]. Overall, 50% of the patients in our study were in the age group of 41-60 years. Sarfraz et al. [11] also reported thyroid cancer to be common in the third, fourth, and fifth decade of life. FTC was common among females with a female-to-male ratio of 1.5: 1 in our study, which is supported by previous studies [12,13]. A total of 24 (60%) patients presented with a complaint of neck swelling, similar to the study conducted by Zuberi et al. [14]. A study from Riyadh, Saudi Arabia reported similar findings of mass/goiter as the most frequent presentation in patients with thyroid cancer reported by three-quarters of their patients [15].

In our study, eight (20%) patients presented with compressive symptoms and metastasis each. In a study by Zuberi et al., 23% of FTC patients had metastasis [14]. Studies by Mizukami et al. and Koppad et al. reported that the primary presentation of FTC may be a metastatic disease [16,17]. The high proportion of cases with distant metastasis at histological diagnosis can be explained by the fact that FTCs have an affinity for blood vessels, which is considered a key diagnostic feature. Patients with metastasis in our study were aged between 40 and 60 years, except for two who were less than 40 years of age. This finding is consistent with a previous study that showed that increasing age and vascular invasion are linked with metastasis [18]. In our study, seven out of eight patients with metastasis had widely invasive FTC. The group of patients with widely invasive FTC had a higher incidence of lymph node involvement and distant metastasis in other studies as well [19,20].

The preoperative thyroglobulin level was tested in six out of eight metastatic cases (75%) in which it was elevated. This emphasizes the importance of checking thyroglobulin level in unusual presentations (bone pain, fractures) of patients with neck swelling to help establish the primary source of the tumor [21].

Radiological features of thyroid nodules are not documented following a standardized pattern, in which suspicious features on sonography should be included. Radiological workup of our patients was done both at our institute and outside. This deficiency needs to be addressed in developing countries by adopting a standardized pattern of documenting a thyroid nodule. In most cases, only the size of the thyroid nodule was mentioned without further details of the echogenicity, calcification, and irregular borders, which can help in classifying patients as low-, intermediate-, and high-risk based on the ultrasound features of the thyroid nodule [6].

On FNAC, 50% (20 patients) had Bethesda category III-IV while 10% (four patients) had category II. According to the ATA guidelines 2015, all categories III and IV should undergo surgery and category II should be monitored [6]. In our study, the ATA guidelines were followed. Among Bethesda category II patients, three developed compressive symptoms while one developed metastasis later, for which thyroidectomy was performed.

Thyroid cancers are mainly treated with surgery while RAI therapy is the main adjuvant therapy as per the ATA guidelines [6]. The same treatment plan was followed in our patients. The neck lymph node dissection was done in 10 patients, of which seven had lymph node involvement on histopathology. The lymph node involvement was almost the same in males and females (18.75% vs. 19.0%). Witte et al. [22] suggested that, although nodal metastasis in FTC is uncommon, when present, it may be an indicator of poor prognosis. Based on these results, we would propose that nodal dissection in FTC be performed only when there is preoperative or intraoperative suspicion of nodal metastases.

On histopathology, 23 (57.5%) patients had minimally invasive FTC while 17 (42.5%) had widely invasive FTC. In other studies, the majority of tumors were also minimally invasive FTC [23,24]. The capsular invasion was present in 39 patients (95%); one patient had no capsular invasion but there was a vascular invasion. Among 17 patients with widely invasive FTC, five were in the age group of 18-40 years and 12 were in the age group of 41-60 years. In our study, 11 out of 17 patients with widely invasive FTC had a tumor size of more than 4 cm. In addition, 13 out of 17 patients with widely invasive FTC had lymphovascular invasion; the record of lymphovascular invasion was not found in four out of 17 widely invasive FTC patients as one patient presented with compressive symptoms and three had surgery done outside and then followed in our center. Seven out of 17 widely invasive FTC patients had metastasis as the initial presentation in our study. One patient had no capsular invasion and was classified as a widely invasive thyroid carcinoma based on widespread vascular invasion. Among the widely invasive FTC patients, nine received RAI at more than 100 mCi, five received 100 mCi, and one patient was advised a high dose of RAI but the family opted to give only 30 mCi; the RAI record for two patients could not be found. These findings are similar to the study by Chow et al., who reported that over 80% of patients underwent postoperative RAI therapy [25]. The maximum dose of RAI received was 700 mCi in a patient with bony metastasis which is almost equal to the maximum dose of RAI in another study by Durante et al. [26].

Among the 23 minimally invasive FTC patients, eight were in the age group of 18-40 years, eight were in the age group of 41-60 years, and seven were in the age group of 61-80 years. In our study, 13 out of 23 minimally invasive FTC patients had lymphovascular invasion, eight patients had no lymphovascular invasion, and the record of two patients was not available as lobectomy was done outside our institution and the patients were admitted for completion thyroidectomy. Their histopathology after completion thyroidectomy showed benign thyroid tissue. One out of 23 minimally invasive FTC cases had metastasis at initial presentation; this patient was a 72-year-old male [27]. Among the minimally invasive FTC patients, five received RAI 30 mCi, three received 100 mCi, and two received 150 mCi. Those receiving more than 100 mCi were in the age group of more than 45 years, had a large tumor size, and displayed metastasis as well as the presence of lymphovascular invasion. Two patients received RAI 30 mCi twice and one patient received 15 mCi RAI instead of 30 mCi. Three patients did not receive RAI due to the low risk of recurrence and young age. RAI records of seven patients could not be found.

In our study, 65% of the patients had clinical stage 1 disease while 25% had stage 2 and 10% had stage 4 disease. All stage IV disease patients were above the age of 55, six stage 2 disease patients were aged 55 years or above, and four were aged less than 55 years who presented with distant metastasis. In stage 1 disease, 22 patients were aged less than 55 years, and only two patients were above the age of 55 years and were classified as stage 1 because they had FTC size less than 4 cm with no extrathyroidal extension, lymphadenopathy, and distant metastasis [28].

This retrospective study has a few limitations. The most important limitations are the small sample size and the fact that it was conducted in a single center.

## Conclusions

FTC can present with both local or metastatic symptoms. The atypical presentation of metastatic FTC should be considered, diagnosed, and managed early to limit mortality and morbidity. Ultrasound is the best diagnostic investigation of choice followed by FNAC. Surgery is the mainstay of treatment and should be followed by RAI in select cases. Thus, understanding the trend of FTC and proper planning and utilization of the resources will help developing countries in effectively treating the FTC.
